# Preoperative neoadjuvant chemotherapy on surgical condition and oncogene expression in advanced gastric cancer

**DOI:** 10.12669/pjms.36.3.1608

**Published:** 2020

**Authors:** Guangyu Sun, Shuyan Wang, Guangsheng Liu

**Affiliations:** 1 Guangyu Sun, Department of Oncology, Binzhou People’s Hospital, Shandong 256600, China; 2 Shuyan Wang, Department of Oncology, Binzhou People’s Hospital, Shandong 256600, China; 3 Guangsheng Liu, Department of Oncology, Binzhou People’s Hospital, Shandong 256600, China

**Keywords:** Advanced gastric cancer, Gene expression, Neoadjuvant chemotherapy, Surgical condition

## Abstract

**Objective:**

To evaluate the effect of preoperative neoadjuvant chemotherapy regimen of XELOX (capecitabine combined with oxaliplatin) on surgical condition and oncogene expression in advanced gastric cancer.

**Methods:**

From January 2015 to July 2016, 124 patients with advanced gastric cancer who were admitted to our hospital were selected. Random number table method was used to divide them into an observation group and a control group, 62 each group. The observation group received two courses of neoadjuvant chemotherapy (XELOX) before operation, and the control group received surgery. The operation condition, expression of oncogenes in gastric cancer lesions, occurrence of adverse reactions and the long-term prognosis were compared between the two groups.

**Results:**

The R0 resection rate of the observation group was significantly higher than that of the control group, and the difference was statistically significant (P<0.05). The operation time of the observation group was shorter than that of the control group, the amount of intraoperative bleeding and the amount of postoperative drainage of the observation group were less than that of the control group, and the differences were statistically significant (P<0.05). The mRNA expression of gastrokine 1, multiple tumor suppressor protein, Wilms tumor gene on the X chromosome (WTX gene) and gene of phosphate and tension homology deleted on chromosome ten (PTEN gene) in the observation group after treatment was significantly higher than that in the control group before treatment, and the increase amplitude of the observation group was more obvious than that of the control group (P<0.05). There was no significant difference in the incidence of adverse reactions between the two groups (P>0.05). In terms of long-term prognosis, the disease-free survival time and average survival time of the observation group during the two-year follow-up period were significantly better than those of the control group, and the recurrence rate of the observation group was significantly lower than that of the control group; the differences were statistically significant (P<0.05).

**Conclusion:**

Preoperative XELOX for advanced gastric cancer patients can effectively increase the proportion of radical surgery, reduce the risk of surgery, and significantly regulate the expression of oncogene, thus improving the long-term prognosis of patients.

## INTRODUCTION

Gastric cancer, one of the most common malignant tumors of digestive system. In recent years, due to the deterioration of the environment and the change of people’s lifestyle, the morbidity and mortality of gastric cancer remain high.^1^,^2^ Early detection and treatment are very important for prolonging the survival of patients with gastric cancer.^3^ However, as early gastric cancer has no specific clinical symptoms and the onset of the disease is hidden, it is usually found when the cancer has developed to the advanced stage. Advanced gastric cancer has strong growth and infiltration activity, and local lesions will have adjacent tissue infiltration and lymph node metastasis. Surgical excision and lymph node dissection can remove visible lesions, but cannot completely remove the cancer cells in small lesions. Some small residual lesions after operation will become the pathological basis of long-term recurrence and metastasis.^4^,^5^

Neoadjuvant chemotherapy refers to systemic chemotherapy before local treatment such as surgery or radiotherapy, which aims at shrinking the mass and killing the invisible metastatic cells as early as possible to facilitate follow-up surgery and radiotherapy.^6^,^7^ A study has shown that preoperative neoadjuvant chemotherapy can effectively remove occult and small lesions and reduce the stage of tumors^8^, which can improve prognosis and prolong survival. However, the influence of preoperative chemotherapy on the surgical condition and malignant features of advanced gastric cancer is not clear in China and abroad, and there is no convincing unified standard for the choice of chemotherapy regimen. The purpose of this study was to explore the effect of XELOX (capacitating combined with oxaliplatin) chemotherapy regimen in preoperative chemotherapy for advanced gastric cancer, in order to provide a theoretical basis for clinical practice.

## METHODS

In this study 124 patients with advanced gastric cancer who were admitted to our hospital were selected. This study was conducted after ethical approval between January 2015 to July 2016. (Ref. No. 141, dated August 3, 2019)

### Inclusion criteria:

1. 18 years old ≤ age ≤ 75 years old.
2. Preoperative pathology indicated primary gastric cancer.
3. Not undergoing treatment such as surgery, radiotherapy and chemotherapy.
4. IIb-IIIcclinical stage of cancer.
5. Karnofsky score ≥60 points.
6. Expected survival time ≥3 months.

### Exclusion criteria:

1. Having other malignant tumors.
2. Being allergic to oxaliplatin and capacitating.
3. Having severe organ dysfunction.
4. Undergoing preoperative chemotherapy.
5. Having severe infectious or autoimmune diseases.

The patients were divided into an observation group and a control group by random number table method, 62 each group. This study was approved by the ethics committee of our hospital. All the selected subjects signed the informed consent.

### Therapeutic method

The observation group was given two courses of XELOX neoadjuvant chemotherapy before operation: capacitating was given twice a day, after breakfast and dinner, at a dose of 2500 mg/m^2^, from d1 to d14, 21 days as a course; oxaliplatin was intravenously dripped on the first day of each course, at a dose of 130 mg/m^2^, and 21 days was taken as a course. The observation group received surgery in the 4^th^ week after the second course of chemotherapy, while the control group received surgery directly. The operation mode of the two groups was consistent. The operation was performed by the same operation team. The operation mode was radical mastectomy combined with D2 lymphadenectomy, and palliative operation was performed for patients who could not undergo radical mastectomy. After operation, adjuvant chemotherapy was given to both groups, for about eight cycles.

### Observation index

### Surgical condition

During the operation, the operation time, intraoperative bleeding volume and postoperative drainage volume were observed, and the R0 resection rate was judged according to the pathological results after resection of the tumours (the resection was complete, and all the incisal edges were negative).

### Gene expression

Gastric cancer lesions which were taken for biopsy were taken before chemotherapy, and gastric cancer lesions which were surgically respected were taken after chemotherapy. RNA in the lesion tissues was isolated and purified using kit (Beijing Cwbio Company, China). Complementary DNA (cDNA) was obtained after performing inverse transcription on RNA, and the reaction system was prepared. The reaction was carried out according to the procedure of pre degeneration at 95°C for three minutes, pre degeneration at 95°C for 25 s, annealing at 60°C for 40 s and extension at 72°C for 35 s. The reaction curve generated in the software after reaction, and the mRNA expression quantity of atropine 1, multiple tumour suppressor protein (p16), Wilms tumour gene on the X chromosome (WTX gene) and gene of phosphate and tension homology deleted on chromosome ten (PTEN gene) was calculated according to the reaction curve.

### Occurrence of adverse reactions

During the recovery, the occurrence of adverse reactions after operation was observed.

### Long-term prognosis

At the end of the two-year follow-up, the long-term prognostic indicators such as disease-free survival time, survival time and recurrence rate were recorded.

### Statistical analysis

All the data were analyzed by SPSS 22.0. The measurement data conformed to normal distribution. The independent sample t-test was used for statistical analysis, and the related data were expressed as Mean±Standard Deviation. The enumeration data were expressed as rate (%). The comparison between groups was performed using X^2^ test. Difference was considered as statistically significant if the value of P was smaller than 0.05.

## RESULTS

There were 32 males and 30 females in the observation group, with an average age of 58.41±12.30 years and body mass index (BMI) of 28.47±7.33 kg/m^2^. As to the pathological stage, there were 15 cases of stage IIb, 11 cases of stage IIIa, 20 cases of stage IIIb, and 16 cases of stage IIIc; as to the location of tumors, there were 31 cases of cardia, 13 cases of pylorus, 16 cases of gastric body, and 2 cases of whole stomach. The control group consisted of 31 males and 31 females, with an average age of 57.31±13.69 years and BMI of 27.88±7.50 kg/m^2^. As to the pathological stage, there were 15 cases of stage IIb, 11 cases of stage IIIa, 20 cases of stage IIIb and 16 cases of stage IIIc. As to the location of tumors, there were 35 cases of cardia, 10 cases of pylorus, 14 cases of gastric body and 3 cases of whole stomach. There was no significant difference in general clinical data between the two groups (P > 0.05); hence the results were comparable.

***:*** The R0 resection rate of the observation group was significantly higher than that of the control group [91.94% (57/62) vs. 66.13% (41/62)], and the difference was statistically significant (X^2^=5.586, P<0.05). The operation time of the observation group was shorter than that of the control group, and the amount of bleeding during operation and the amount of drainage after operation were less than that of the control group; the differences were statistically significant (P<0.05, Table-I).

**Table-I:** Surgical conditions between the two groups.

Group	Operation time (min)	Intraoperative bleeding (mL)	Postoperative drainage volume (mL)
Observation group	139.27±16.60	259.74±52.69	198.47±48.55
Control group	151.86±18.04	291.31±61.39	231.75±59.91
T	4.217	3.169	3.516
P	0.024	0.031	0.034

The expression quantity of motilin 1, p16, WTX and PTEN genes in both groups was significantly higher after chemotherapy compared to before chemotherapy, and the increase amplitude of the observation group was significantly higher than that of the control group; the differences had statistical significance (P<0.05, Table-II).

**Table-II:** Oncogene expression in lesions between two groups.

Group		Motilin 1	P16	WTX gene	PTEN gene
Observation group	Before treatment	1.01±0.12	1.01±0.11	1.04±0.15	0.99±1.03
	After treatment	1.90±0.21*^#^	2.24±0.31*^#^	2.03±0.29*^#^	1.95±0.22*^#^
Control group	Before treatment	0.98±0.15	1.02±0.13	0.99±1.02	1.01±0.12
	After treatment	1.43±0.15*	1.61±0.22*	1.56±0.19*	1.43±0.18*

***Note:***

* indicated P<0.05 compared to the same group before treatment,

# indicated P<0.05 compared to the control group.

The incidence of adverse reactions during treatment in the observation group was 33.3%, and there was no significant difference with the control group (27.8%, P>0.05, Table-III).

**Table-III:** Adverse reactions between the two groups (%).

Group	Few platelets	Reduced leukopenia	Nausea and vomiting	Diarrhea	Total complications
Observation group	4(6.45)	3(4.84)	6(9.68)	7(11.29)	20(32.26)
Control group	6(9.68)	4(6.45)	5(8.06)	2(3.23)	17(27.42)
X^2^	/	/	/	/	0.257
P	/	/	/	/	0.017

During the two-year follow-up, the disease-free survival time and average survival time of the observation group were significantly better than those of the control group, and the recurrence rate of the observation group was significantly lower during the follow-up period; the difference were statistically significant (P<0.05, Table-IV).

**Table-IV:** Long-term prognosis of two groups of patients.

Group	Disease-free survival (months)	Survival time (months)	Recurrence rate (%)
Observation group	18.88±2.04	20.38±3.24	13(20.97)
Control group	15.61±2.22	17.08±3.50	27(43.55)
X^2^/t	9.257	5.654	7.782
P	0.001	0.014	0.007

## DISCUSSION

In recent years, as the study of the biological characteristics of gastric cancer goes deeper, the chemical therapy for gastric cancer has made remarkable progress, but surgery is still the most important treatment for gastric cancer. However, surgery cannot change the local recurrence and long-term metastasis characteristics of residual cancer cells. The advanced gastric cancer patients especially have more than 60% of postoperative recurrence and metastasis rate, which severely reduces the post-operative rehabilitation effect.^9^-^11^ A study shows that adjuvant chemotherapy is helpful in killing micro-metastatic lesions^12^, so a new treatment model of surgical resection of cancer after neoadjuvant chemotherapy has gradually formed.

The results of this study suggested that preoperative XELOX for advanced gastric cancer could improve the resection rate of radical mastectomy, reduce intraoperative bleeding and postoperative drainage, and enhance the long-term prognosis of patients with advanced gastric cancer. Capecitabine is an oral form of fluorouracil chemotherapeutic drug in XELOX regimen. 5-FU is the most effective chemotherapeutic drug for digestive tract tumours. It has long been considered as the cornerstone of chemotherapy for digestive tract tumours.^13^,^14^ Oxaliplatin as a third-generation platinum chemotherapeutic drug combined with fluorouracil’s has been found being significantly effective in improving the chemotherapeutic efficacy of advanced gastric cancer.^15^,^16^ Tian’s study found that pre-operative XELOX regimen combined with chemotherapy could improve the radical resection rate of gastric cancer and reduce the clinical stage of cancer to improve the long-term prognosis of patients,^17^ which is consistent with the results of this study. The advantages of preoperative adjuvant chemotherapy lie in increasing the concentration of chemotherapeutic drugs in tumour tissue before blocking the blood supply of tumour tissue to increase the effect of chemotherapy and kill tumour cells to the greatest extent; reducing the activity of tumour cells, reducing the spread of iatrogenic tumours in the course of surgery, and inactivating small lesions that cannot be involved in surgery, thereby reducing the recurrence and metastasis rate of tutor after surgery.^18^,^19^

In addition, there was no significant difference in the incidence of adverse reactions between the two groups (P>0.05). The reason might be that the operation was performed at the 4th week after two cycles of preoperative chemotherapy, during which the physical condition and immunity of the patients recovered basically. It suggested that neoadjuvant chemotherapy was safe. However, Li et al. pointed out that neoadjuvant chemotherapy might increase the risk of surgery and lead to related postoperative complications.^20^ This result is different from that in this study, which may be related to the size of the sample; therefore, it needs further study and verification.

The over proliferation and invasion of gastric cancer cells are closely related to the activation of proto-ontogenesis and the inactivation of various anti-ontogenesis. Motilin 1 encodes a class of gastric mucosal protective proteins, which can repair and protect gastric mucosa, and its loss of expression will increase the damage of carcinogenic factors to gastric mucosal cells and promote malignant transformation.^21^ P16 is a negative regulatory molecule of cell cycle, which can hinder the formation of multiple Cyclin complexes to induce cell cycle arrest.^22^ WTX and PTEN are antagonists of Wnt pathway and protein kinase B pathway, and cell apoptosis will happen after the growth promoting effect of the above pathways are blocked.^23^

By analyzing the changes of proto-oncogene expression in gastric cancer lesions, it was found that the mRNA expression quantity of motilin-1, p16, WTX and PTEN genes in gastric cancer lesions of the observation group increased more significantly than that of the control group. The result suggests that neoadjuvant chemotherapy before operation has a more prominent effect on tumour suppressor gene than radical operation alone. This result is also rarely reported in previous studies.^24^

### Limitations of the study

This study is a single-center, small-sample study, with a low level of evidence. How to maximize the advantages of preoperative chemotherapy and improve the long-term prognosis of patients still need to be further explored.

## CONCLUSION

Preoperative radiotherapy combined with neoadjuvant chemotherapy can improve the resection rate of advanced gastric cancer without increasing the incidence of adverse reactions. At the same time, combined therapy can significantly increase the expression of anti-ontogeny and prolong the survival of patients, which is worthy of promotion.

### Authors’ Contribution:

**GYS:** Study design, data collection and analysis and is responsible and accountable for the accuracy or integrity of the work.

**GYS & SYW:** Manuscript preparation, drafting and revising.

**GYS & GSL:** Review and final approval of manuscript.
